# Effects of Abdominal Microcurrent in the Consumption and Proportion of Energy Substrates during Aerobic Exercise: A Pilot Study

**DOI:** 10.3390/healthcare10050917

**Published:** 2022-05-16

**Authors:** Rui Vilarinho, Susana Miriam Faria, Pedro Ribeiro Rocha Monteiro, Cristina Melo, Rubim Santos, Andreia Noites

**Affiliations:** 1 Center for Rehabilitation Research, School of Health, Polytechnic Institute of Porto, 4200-072 Porto, Portugal; pmonteiro@ess.ipp.pt (P.R.R.M.); cam@ess.ipp.pt (C.M.); rss@ess.ipp.pt (R.S.); arn@ess.ipp.pt (A.N.); 2 Department of Physiotherapy, School of Health, Polytechnic Institute of Porto, 4200-072 Porto, Portugal; susana.miriam.faria@gmail.com; 3 Department of Functional Sciences, School of Health, Polytechnic Institute of Porto, 4200-072 Porto, Portugal; 4 Department of Physics, School of Health, Polytechnic Institute of Porto, 4200-072 Porto, Portugal

**Keywords:** lipids, adipose tissue, exercise prescription, indirect calorimetry, obesity

## Abstract

Microcurrent therapy can increase lipolytic activity. However, it is unknown if the increased availability of lipids can influence the selection of energy substrates during a single session of aerobic exercise. We aimed to analyze the effect of microcurrent application to the abdominal region in the consumption of lipids and carbohydrates, and respiratory exchange ratio (RER) during a single session of moderate aerobic exercise in young adults. A pilot study was conducted in which participants were allocated to intervention (IG) or placebo (PG) groups. In both groups, 40 min of microcurrent application with two frequencies (25 and 10 Hz) followed by 50 min of moderate-intensity aerobic exercise (45–55% of heart rate reserve) on a cycloergometer were performed. The microcurrent application was performed without intensity in the PG. A portable gas analyzer (K4b^2^) was used during exercise in both groups. Thirty-eight participants (20.6 ± 1.8 years; 18 in IG and 20 in PG) were enrolled. There were no significant differences in the consumption of substrates or RER between the groups during exercise (*p* > 0.05). Microcurrent application seems to be insufficient to influence the consumption of energy substrates and RER during a single session of aerobic exercise in young adults.

## 1. Introduction

The main cause of weight gain that subsequently leads to obesity is the positive energy balance resulting from increased energy intake and insufficient physical activity (PA) [1]. Obesity is characterized by an accumulation of energy in the adipose tissue (AT) in the form of triglycerides (TG) [2], and cumulative positive energy balances can result in a saturation of AT expansion capacity and consequent adipocyte dysfunction [3]. Excessive abdominal fat (abdominal obesity), especially the accumulation of visceral adipose tissue (VAT), is more associated with adipocyte dysfunction, leading to systemic low-grade inflammation, ectopic lipid deposition, and reduced insulin sensitivity [4]. These effects increase the risk of several diseases, including type 2 diabetes mellitus, and cardiovascular disease, among other diseases [5,6,7].

Lifestyle modification is the main intervention for managing obesity [8]. Weight loss can be achieved by increasing energy expenditure (exercise training) and reducing calory intake (diet), resulting in a negative energy balance. In particular, energy expenditure during exercise training is one of the important strategies for lifestyle modification. This depends on the type of exercise, as well as its intensity and duration. During exercise training involving aerobic exercise with low-to-moderate intensity and of long duration, fat is the major source of energy and, therefore, such exercise is one of the best options to lose fat mass [9,10].

Although exercise training can reduce fat mass, in recent years microcurrent therapy have been associated with it to reinforce fat mass reduction, especially in the abdominal region. Microcurrent therapy is an electrotherapy option characterized by the application of a low-frequency and low-intensity current across the skin without muscle contractions and perceptible sensations, and its potential mechanism is different from that of conventional transcutaneous electrical nerve stimulation [11]. In clinical practice, this option of electrotherapy is mainly used for the treatment of musculoskeletal conditions [12]. Recently, microcurrent therapy has been applied in clinical practice based on studies that showed that electrical stimulation has a potential effect on the activation of lipolysis in adipocytes in vitro [13]. Posteriorly, studies have explored the combined use of microcurrent application and exercise training with promising results [14,15]. More specifically, studies that applied a combined protocol of microcurrent application in the abdominal region and moderate aerobic exercise showed a higher lipolytic activity, compared to exercise alone, in young healthy subjects and people with coronary artery disease [16,17,18]. These findings are important when considering the association of microcurrent application with exercise training, since the increase of free fatty acids in blood circulation resulting from increased lipolysis activity may become available for oxidation and energy expenditure during an exercise training session. However, to the best of our knowledge, it is currently unknown whether this increasing availability of fatty acids can influence the selection of energy substrates during the first exercise training session.

Therefore, the aim of this study was to analyze the effect of a single session of microcurrent application in the abdominal region on the consumption of energy substrates (lipids and carbohydrates), and respiratory exchange ratio (RER) values during a single session of moderate aerobic exercise in young adults. It was also an aim of this study to analyze the effect of the same protocol on the consumption of energy substrates and RER values according to sex. We hypothesized that microcurrent application may increase the consumption of lipids and, consequently, lower RER values would be observed. These results may be more pronounced in women since the contribution of energy substrates for energy expenditure is influenced by sex, especially on fat metabolism.

## 2. Materials and Methods

### 2.1. Study Design, Participants

A double-blind, pilot study was conducted in subjects aged 18 years or above in both sexes recruited from a campus university. Exclusion criteria were: (i) pregnant women or postpartum in the last year; (ii) subjects with smoking or drinking habits; (iii) the presence of metabolic, digestive, hematologic, renal, cardiac, respiratory, neurological, or musculoskeletal diseases; (iv) subjects with skin alterations, especially in the abdominal region; (v) subjects with electronic or metallic devices (such as pacemakers); (vi) subjects undertaking a weight loss program.

Eligible subjects were identified and contacted by the researchers, who explained the purpose of the study and asked about their willingness to participate. Participants who accepted enrolment in the study were randomly assigned to the intervention group (IG) or placebo group (PG). A distributed randomization process was performed in Microsoft Excel.

Approval for this study was obtained from the ethics committee of the School of Health—Polytechnic Institute of Porto (protocol code 04131). This study was registered at ClinicalTrials.gov (registry number NCT02110927). Written informed consent was obtained from all participants before any data collection.

### 2.2. Sample Size

According to the recommendations for adequate sample sizes to conduct pilot studies, at least twelve participants in each group were required to conduct the study [19].

### 2.3. Intervention

The study was performed between January and May of 2015. Data collection for each participant lasted one day with a total time of three hours. All data collection for all participants was made in the morning. Specific instructions were given to the participants to avoid physical activity and any intake of caffeine and alcohol in the 24 h prior to study, get at least 8 h of sleep the night before, to eat a light meal and to ingest 500 mL of water in the two hours before the tests [18].

**Figure 1:**
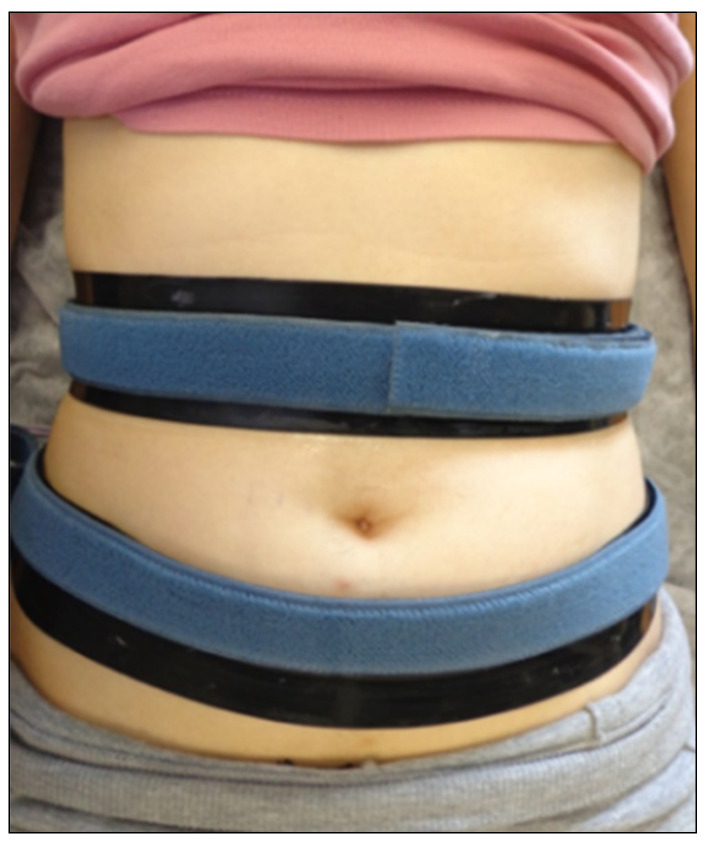
Placement of the transcutaneous electrodes in a band in the abdominal region.

The intervention consisted of non-invasive microcurrent application in the abdominal region followed by moderate aerobic exercise in both groups. The microcurrent was applied to participants in the supine position with an Enraf Nonius^®^ device, model Sonopuls 692 (The Netherlands), using transcutaneous electrodes in a band with a conductive gel, with a distance of 5–10 cm between electrodes (Figure 1).

A 25 Hz frequency (impulse duration and resting duration of 20 msec) was used during the first 20 min, followed by another application of 20 min at 10 Hz frequency (impulse duration and resting duration of 50 msec) [16,17,18,20]. The microcurrent intensity was set at 1 milliampere. In PG, all the procedures were similar to IG but the microcurrent device was turned off without the participants knowing. The researcher responsible for the microcurrent protocol was not responsible for the assessment of outcomes and the aerobic exercise protocol.

After completing the application of the microcurrent, participants performed aerobic exercise on a cycloergometer (Monark, model 928E, Sweden) and a portable gas analyzer K4b^2^ (COSMED^®^) was used to analyze oxygen consumption (VO_2_), carbon dioxide production (VCO_2_), and to determine the RER during three minutes at rest. Immediately after, participants performed aerobic exercise, also with continuous analysis using the K4b^2^. The K4b^2^ system proved to be an effective in assessing both VO_2_ and the VCO_2_ in a wide range of exercise intensities, presenting values to the limits of agreement and bias for minute ventilation (VE) of ±1.63 and ±1.27 L/min, for VO_2_ of ±0.82 and ±0.08 L/min and for the VCO_2_ of ±0.67 and ±0.06 L/min, respectively. The ICC values were VE: 0.58–0.78; VO_2_: 0.53–0.87; VCO_2_: 0.68–0.81 [21,22,23]. The standard calibrations of the K4b^2^ were guaranteed during the study. The aerobic exercise protocol was characterized by a 50 min duration and a moderate intensity (45 and 55% of HR reserve) [9,10] monitored by heart rate (HR) using a cardiofrequencimeter (model FT7, POLAR^®^, USA). For this purpose, the assessment of resting HR on each participant was performed, where participants were kept in a sitting position for 5 min and the resting HR value was recorded at the moment of its stabilization. Afterwards, a calculation of the theoretical maximum HR (HR_max_) using the equation developed by Tanaka et al. (2001) [24] was conducted:

HR_max_ = 208 − (0.7 × age), (1)

Through this calculation, and knowing the resting HR, the next step was to calculate the target HR corresponding to an intensity between 45 and 55% of HR reserve (HR_reserve_), using the Karvonen formula [25]:

Target HR = resting HR + %intensity (HRmax − resting HR),(2)

The aerobic exercise protocol consisted of: (a) a warm-up (5 min) in which the work rate was gradually raised (initial work rate—50 W for females and 75 W for males) [25] as well as the pedal revolutions per minute (target: 60 rpm), until reaching the target HR (45–55% HR_reserve_); (b) body (45 min) during which the target HR was maintained at 60 rpm; (c) Cool-down (5 min) during which HR was gradually reduced.

### 2.4. Measurements

Sociodemographic data (age, sex), anthropometric data (height, weight, body mass index [BMI], total body fat percentage [%TBF], waist circumference [WC] and waist-to-height ratio [WHtR]), PA levels, and dietary intake were collected. Percentage TBF was collected with bioelectrical impedance using a Tanita BC-545 N (Tanita, Amsterdam, The Netherlands). Waist circumference was taken with the participants in a standing position at the approximate midpoint between the lower margin of the last palpable rib and the top of the iliac crest [26]. The waist-to-height ratio was calculated as waist circumference divided by height.

PA levels were assessed with the short form of the International Physical Activity Questionnaire (IPAQ-SF), which has been shown to have good reliability and validity for the Portuguese population [27]. Participants were asked about duration (minutes) and frequency (days) of walking, moderate and vigorous intensity activities in the last seven days. A combined total PA score (MET-min/week) was calculated using IPAQ orientations (available at www.ipaq.ki.se, accessed on 5 April 2015).

A semi-quantitative Food Frequency Questionnaire, referring to 12 months prior to the study, was used to monitor dietary intake. All participants answered the questionnaire on the day they participated in the study. The Food Processor Plus^®^ (ESHA Research, Salem, Oregon) was used to convert food into nutrients (proportion of protein, carbohydrates, and total fat, and total of calories intake) [28].

#### Substrate Oxidation and RER Values

Based on the K4b^2^ data recorded, substrate oxidation (lipids and carbohydrates) and RER were obtained and averaged from the 3-min rest period (Rest) and every 5-min period during the entire 50 min of exercise (warm-up, [0–5[, [5–10[, [10–15[, [15–20[, [20–25[, [25–30[, [30–35[, [35–40[ and cool-down). The rates of substrate oxidation, expressed in grams per minute (g/m), were calculated according to the following equations [29]:

Lipids (g/min) = (1695 × VO_2_) − (1701 × VCO_2_), (3)

Carbohydrates (g/min) = (4585 × VCO_2_) − (3226 × VO_2_), (4)

with gas volumes expressed in liters per minute. These equations are based on the assumption that protein breakdown contributes little to energy metabolism during exercise [30].

**Figure 2:**
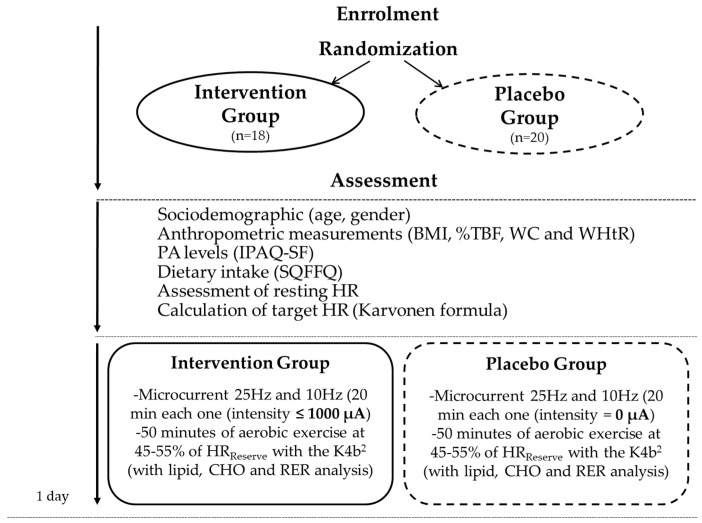
Overview of the procedures of the study. BMI, body mass index; %TBF, total body fat percentage; WC, waist circumference; WHtR, waist-to-height ratio; IPAQ-SF, short form of the international physical activity questionnaire; SQFFQ, Semi-quantitative Food Frequency Questionnaire; HR, heart rate; CHO, carbohydrate; RER, respiratory exchange ratio.

An overview of the procedures of the study is shown in Figure 2.

### 2.5. Statistical Analysis

Statistical analyses were performed using IBM SPSS Statistics version 24.0 (IBM Corporation, Armonk, NY, USA). The level of significance was set at 0.05.

Descriptive statistics (mean ± standard deviation) were used to describe each sample.

The normality of the data was determined with the Shapiro-Wilk test. Then, independent *t* tests were used to compare sociodemographic, anthropometric, PA levels and dietary intake between groups (IG and PG). The same test was used to compare the substrate oxidation (lipids and CHO) and RER during rest between groups and according to sex (females from IG vs. females from PG and males from IG vs. males from PG).

To compare the substrate oxidation (lipids and CHO) and RER during exercise between the groups, we used repeated-measure ANOVAs. When appropriate, the Bonferroni post hoc test was performed to delineate at which points statistical significance was reached. The same tests were used to compare the groups according to sex (females from IG vs. females from PG and males from IG vs. males from PG) during exercise.

## 3. Results

**Figure 3:**
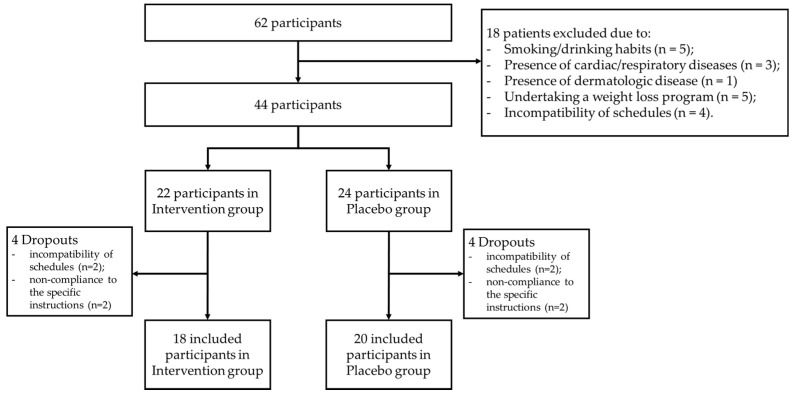
Flow diagram of participants through the study.

Sixty-two participants volunteered for possible inclusion in the study. From these, eighteen were excluded due to smoking/drinking habits (*n* = 5), the presence of cardiac/respiratory diseases (*n* = 3), the presence of dermatologic disease (*n* = 1), undertaking a weight loss program (*n* = 5), and incompatibility of schedules (*n* = 4). Thus, 44 participants were invited to participate in the study and allocated to either the IG or PG. Four participants in the IG (two due to incompatibility of schedules and two to non-compliance to the specific instructions) and four in the PG (two due to incompatibility of schedules and two to non-compliance to the specific instructions) dropped out of the study. Therefore, thirty-eight participants (20 females, 20.6 ± 1.8 years), eighteen in the IG (nine females and nine males) and 20 in the PG (11 females and 9 males), were included (Figure 3).

Participants’ characteristics are summarized in Table 1. No significant differences were observed between groups at baseline (*p* > 0.05).

Participants’ characteristics according to sex are summarized in Table 2. Only one significant difference was observed in %TBF in males between groups (IG vs. PG) (*p* = 0.038). However, both mean values were normal [31].

### 3.1. Lipid and Carbohydrate Consumption

**Figure 4:**
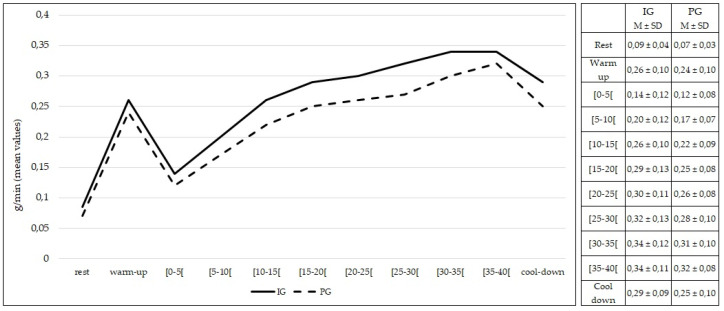
Lipid oxidation during rest, exercise, and cool-down in intervention group and placebo group. Data are expressed as mean ± standard deviation. IG: intervention group; PG: placebo group.

The rate of lipid oxidation at rest was not different between the IG and PG (*p* > 0.05). No differences (*p* > 0.05) were found in the rate of lipid oxidation during the entire 50 min of exercise between the IG and PG (Figure 4).

**Figure 5:**
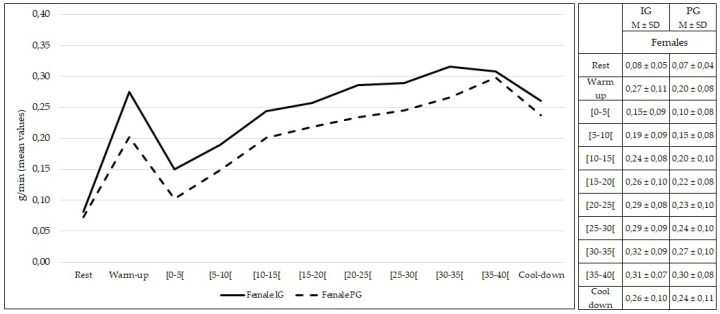
Lipid oxidation during rest, exercise, and cool-down in females (intervention group vs. placebo group). Data are expressed as mean ± standard deviation. IG: intervention group; PG: placebo group.

**Figure 6:**
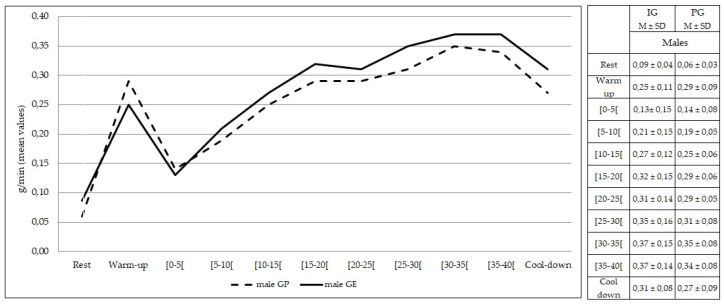
Lipid oxidation during rest, exercise, and cool-down in males (intervention group vs. placebo group). Data are expressed as mean ± standard deviation. IG: intervention group; PG: placebo group.

Comparing females from both groups, no statistically significant differences were found at rest and during exercise (*p* > 0.05) in the rate of lipid oxidation (Figure 5). The same findings were observed comparing males from both groups (*p* > 0.05) (Figure 6).

**Figure 7:**
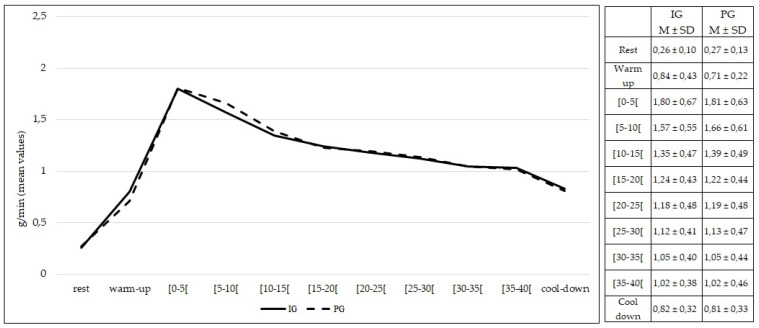
Carbohydrate oxidation during rest, exercise, and cool-down in intervention group and placebo group. Data are expressed as mean ± standard deviation. IG: intervention group; PG: placebo group.

The rate of carbohydrate oxidation at rest was not different between the IG and PG (*p* > 0.05). No differences (*p* > 0.05) were found in the rate of carbohydrate oxidation during the entire 50 min of exercise between the IG and PG (Figure 7).

**Figure 8:**
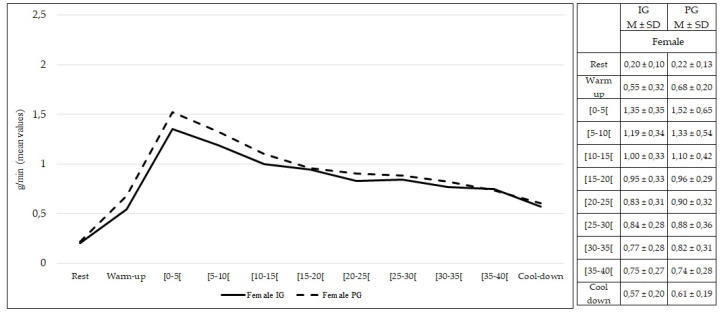
Carbohydrate oxidation during rest, exercise, and cool-down in females (intervention group vs. placebo group). Data are expressed as mean ± standard deviation. IG: intervention group; PG: placebo group.

**Figure 9:**
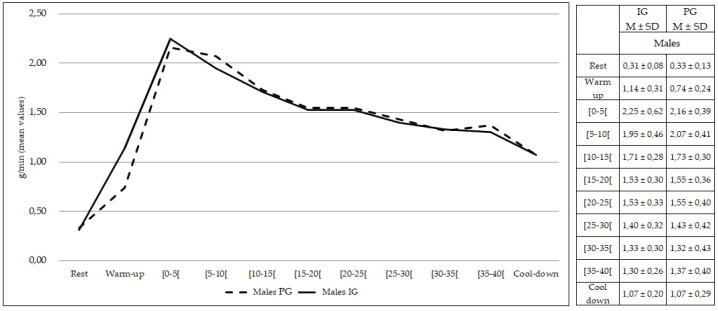
Carbohydrate oxidation during rest, exercise, and cool-down in males (intervention group vs. placebo group). Data are expressed as mean ± standard deviation. IG: intervention group; PG: placebo group.

Comparing females from both groups, no statistically significant differences were found at rest and during exercise (*p* > 0.05) in rate of carbohydrate oxidation (Figure 8). The same findings were observed comparing males from both groups (*p* > 0.05) (Figure 9).

### 3.2. Substrate Energy Proportion—Respiratory Exchange Ratio

**Figure 10:**
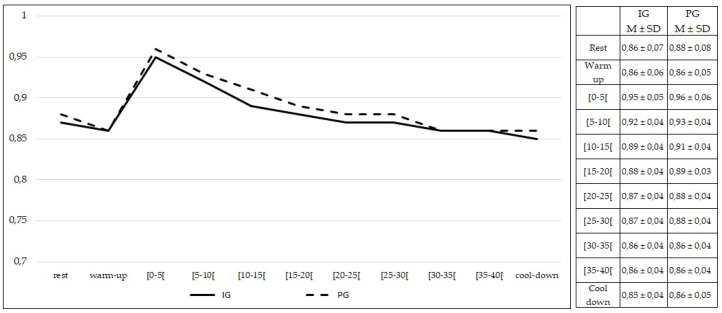
Respiratory exchange ratio values during rest, exercise, and cool-down in intervention group and placebo group. Data are expressed as mean ± standard deviation. IG: intervention group; PG: placebo group.

RER values at rest were not different between the IG and PG (*p* > 0.05). No differences (*p* > 0.05) were found in the RER values during the entire 50 min of exercise between the IG and PG (Figure 10).

**Figure 11:**
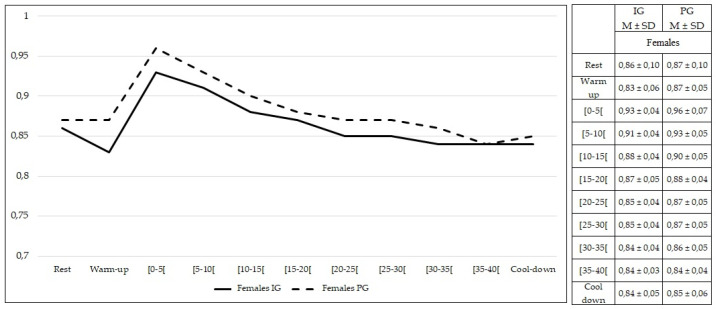
Respiratory exchange ratio values during rest, exercise, and cool-down in females (intervention group vs. placebo group). Data are expressed as mean ± standard deviation. IG: intervention group; PG: placebo group.

**Figure 12:**
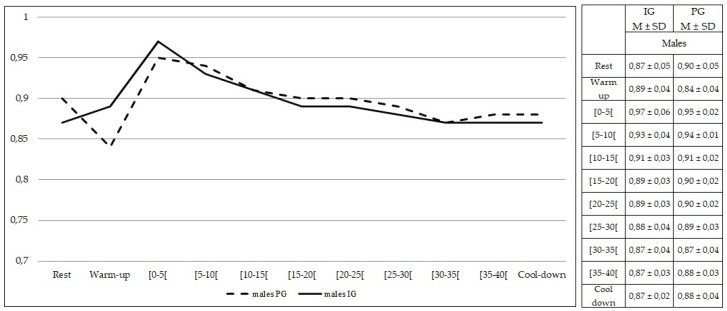
Respiratory exchange ratio values during rest, exercise, and cool-down in males (intervention group vs. placebo group). Data are expressed as mean ± standard deviation. IG: intervention group; PG: placebo group.

Comparing females from both groups, no statistically significant differences were found at rest and during exercise (*p* > 0.05) in RER values (Figure 11). The same findings were observed comparing males from both groups (*p* > 0.05) (Figure 12).

## 4. Discussion

This study analyzed the effect of a single session of microcurrent application in the abdominal region in the consumption of energy substrates (lipids and carbohydrates) and RER values during a single session of moderate aerobic exercise in young adults. It was also an aim of this study to analyze the effect of the same protocol in the consumption of energy substrates and RER values according to sex. For this purpose, the groups in our study (IG and PG) showed similar characteristics, namely age, anthropometric measures, PA levels and dietary intake, which reinforce the comparison between groups in our primary outcome measures and the control of factors that influence metabolism [32].

To the best of our knowledge, this is the first study that analyzes the influence of microcurrent application in the selection of energy substrates during exercise.

The results of this study suggest that microcurrent application did not influence the consumption of lipids and carbohydrates, and RER values during the session of moderate aerobic exercise, since no significant differences were found between groups. These findings suggest that a single session of microcurrent application is not sufficient to increase the selection of lipids and, subsequently, the observation of lower RER values during exercise. This assumption was based on the results obtained in our last clinical trial, where we observed in 83 participants that a single session of microcurrent application in the abdominal area associated with a single session of moderate aerobic exercise significantly increased the glycerol mobilization (as a lipolysis marker), compared to an isolated moderate aerobic exercise session [18]. These results were important considerations in the study of the influence of microcurrent application on the consumption of energy substrates and RER values during exercise, since its action in increasing lipolysis activity and, subsequently, increase of free fatty acids in the blood circulation, allows greater availability of this energy substrate for oxidation and energy expenditure. Therefore, more studies with more participants and with more sessions of microcurrent application and aerobic exercise are important to analyze and explore the dose-effect relationship of this combined intervention in the consumption and proportion of energy substrates. The inclusion of participants with an overweight condition is also important to compare the results with participants having normal body mass.

Microcurrent application has been studied as an important therapy for greater lipolytic activity based on the study conducted by Hamida et al. (2011) in which they studied the effect of electrical stimulation in human adipocytes in vitro, finding an activation of lipolysis through the estimation of the released glycerol [13]. The same study was based on previous studies conducted by Ramirez-Ponce et al. (1998, 2003), in which they concluded that adipocytes have voltage-dependent potassium channels (K) involved in lipolytic activity. These findings suggest a possible lipolytic pathway through microcurrent application that might involve voltage-dependent K channels, with stimulation of the adrenergic activity (with a possible increase in circulating catecholamines) and, subsequently, stimulation of the lipolytic cascade [33,34]. Therefore, due to this possible action of microcurrent application on the sympathetic-adrenergic pathway, its influence on catecholamine concentrations (and their metabolites) through chromatography is also recommended in future studies.

One of the main questions raised during the development of this study was the possible influence of microcurrent application on the quantity of glucose, with a possible decrease in its contribution during aerobic exercise due to the increased availability of lipids, since one of the most important factors in selecting the energy substrates for oxidation in healthy individuals during exercise is their availability in blood circulation [35,36,37,38]. In this study, glucose was the main substrate (quantitatively) during aerobic exercise, but no significant differences were found between groups, or according to sex.

According to our second aim, no significant differences were found in the consumption of energy substrates and RER values between females of IG and PG. The same findings were observed for males. This aim was proposed because the contribution of energy substrates for energy expenditure is influenced by sex, especially fat metabolism, due to different body compositions [25] and differences in the adrenergic stimulation of lipolysis [39,40]. Females seem more able to use lipids as an energy substrate [41,42,43] due to their higher quantity of AT (proven by the mean values of %TBF observed in our results), so we assumed that the effect of microcurrent application on lipolytic activity could have an additional effect on lipid consumption in female in the IG. Females also have a higher number of β-adrenergic receptors (which contributes to lipolysis activity) in AT in the abdominal region compared to males [39]. Knowing that microcurrent can be involved in the adrenergic activity, with a possible increase in circulating catecholamines, we presumed that the application of microcurrents could also result in a higher lipid consumption on females of the IG due to the higher stimulation of β-receptors and, subsequently, higher lipolytic activity. However, more studies with more participants of both sexes are important to confirm these hypotheses.

Some limitations of the present study should be addressed. The need for female participants to undertake the study in the same menstrual phase is a limitation, as well as the control of ingestion of oral contraceptives. However, it is unknown how much the variation of substrates oxidation can be attributed to these factors [44,45,46,47]. Another limitation is the lack of application of a graded exercise test to exhaustion to assess cardiorespiratory fitness (VO_2_max) [25] to ensure similar levels between groups. In addition, this graded exercise test to exhaustion would have allowed a valid prescription of the moderate intensity of aerobic exercise (%VO_2_max), by determining the intensity that elicits maximal fat oxidation (Fatmax) [35].

## 5. Conclusions

The results of this study show that a single session of microcurrent application in the abdominal region seems to be insufficient to influence the consumption of energy substrates (lipids and carbohydrates) and RER values during a single session of moderate aerobic exercise in young adults of both sexes.

## Figures and Tables

**Table 1:** Baseline characteristics of participants.

Characteristics	All Participants (*n* = 38)	Intervention Group (*n* = 18)	Placebo Group (*n* = 20)	*p* Value between Groups
Age (years)	20.6, ± 1.8	20.7 ± 1.8	20.6 ± 1.9	0.845
Height (m)	1.7 ± 0.1	1.7 ± 0.1	1.7 ± 0.1	0.830
Body mass (kg)	66.8 ± 10.9	66.9 ± 6.3	66.8 ± 11.7	0.430
BMI (kg/m^2^)	23.1 ± 2.8	24.0 ± 2.8	22.3 ± 2.6	0.060
%TBF	19.9 ± 8.8	22.4 ± 7.6	16.6 ± 4.3	0.108
WC (cm)	79.6 ± 8.1	80.0 ± 4.9	77.5 ± 5.8	0.156
WHtR	0.5 ± 0.1	0.5 ± 0.1	0.4 ± 0.1	0.060
IPAQ-SF (MET/min/week)	3447.0 [1989; 3958]	3425.0 [1612; 4196]	3453.0 [2272; 3447]	0.613
Calories ^1^	2348.1 ± 669.4	2242.4 ± 745.4	2443.3 ± 596.2	0.363
% Protein ^1^	22.4 ± 3.4	22.0 ± 2.4	22.5 ± 2.8	0.826
% CHO ^1^	60.0 ± 5.7	61.0 ± 4.3	60.9 ± 4.4	0.693
% Total fat ^1^	17.6 ± 3.6	17.0 ± 2.5	16.6 ± 1.7	0.589

Data are expressed as mean ± standard deviation or median [interquartile range]. ^1^ Data obtained from the semi-quantitative food frequency questionnaire. BMI, body mass index; %TBF, total body fat percentage; WC, waist circumference; WHtR, waist-to-height ratio; IPAQ-SF, short form of the international physical activity questionnaire, CHO carbohydrate.

**Table 2:** Baseline characteristics of participants according to sex.

Characteristics	Intervention Group (*n* = 18)		Placebo Group (*n* = 20)		*p* Value between Groups	
	Females (*n* = 9)	Males (*n* = 9)	Females (*n* = 11)	Males (*n* = 9)	Females	Males
Age (years)	20.3 ± 2.3	21.0 ± 1.0	20.2 ± 1.6	21.0 ± 2.2	0.846	1.000
Height (m)	1.63 ± 0.1	1.75 ± 0.1	1.62 ± 0.1	1.80 ± 0.0	0.598	0.480
Body mass (kg)	62.2 ± 3.3	68.7 ± 8.5	53.7 ±8.3	72.8 ± 6.5	0.149	0.965
BMI (kg/m^2^)	24.1 ± 1.9	22.9 ± 1.6	20.9 ± 2.6	22.0 ± 1.5	0.090	0.310
%TBF	29.3 ± 4.0	14.3 ± 3.1	19.7 ± 5.8	11.8 ± 3.8	0.053	0.038 *
WC (cm)	80.0 ± 5.2	83.4 ± 9.7	75.2 ± 9.4	80.8 ± 5.0	0.176	0.480
WHtR	0.49 ± 0.0	0.48 ± 0.1	0.46 ± 0.0	0.45 ± 0.0	0.140	0.233
IPAQ -SF (MET/min/week)	3447.0 [647.0; 4173.0]	3420.0 [2536.0; 5424.0]	2772.0 [1035.0; 3324.0]	3447.0 [3447.0; 3963.0]	0.766	0.863
Calories ^1^	2261.7 ± 298.1	1851.7 ± 773.4	2348.1 ± 752.5	2330.1 ± 320.5	0.569	0.270
% Protein ^1^	20.8 ± 2.5	21.3 ± 2.3	22.4 ± 1.9	20.6 ± 2.7	0.849	0.507
% CHO ^1^	61.0 ± 4.1	61.0 ± 6.3	60.4 ± 4.7	62.8 ± 4.8	0.909	0.627
% Total fat ^1^	18.2 ± 2.8	17.7 ± 2.7	17.2 ± 2.1	16.6 ± 1.4	0.470	0.627

Data are expressed as mean ± standard deviation or median [interquartile range]. ^1^ Data obtained from the semi-quantitative food frequency questionnaire. BMI, body mass index; %TBF, total body fat percentage; WC, waist circumference; WHtR, waist-to-height ratio; IPAQ-SF, short form of the international physical activity questionnaire, CHO carbohydrate. * *p* < 0.05.

## References

[1] Kolnes K.J., Petersen M.H., Lien-Iversen T., Højlund K., Jensen J. (2021). Effect of Exercise Training on Fat Loss—Energetic Perspectives and the Role of Improved Adipose Tissue Function and Body Fat Distribution. Front. Physiol..

[2] Arner P., Bernard S., Salehpour M., Possnert G., Liebl J., Steier P., Buchholz B.A., Eriksson M., Arner E., Hauner H. (2011). Dynamics of human adipose lipid turnover in health and metabolic disease. Nature.

[3] Lafontan M. (2014). Adipose tissue and adipocyte dysregulation. Diabetes Metab..

[4] Virtue S., Vidal-Puig A. (2010). Adipose tissue expandability, lipotoxicity and the Metabolic Syndrome—An allostatic perspective. Biochim. Biophys. Acta (BBA)-Mol. Cell Biol. Lipids.

[5] Unger R.H., Clark G.O., Scherer P.E., Orci L. (2010). Lipid homeostasis, lipotoxicity and the metabolic syndrome. Biochim. Biophys. Acta (BBA)-Mol. Cell Biol. Lipids.

[6] Wang S., Soni K.G., Semache M., Casavant S., Fortier M., Pan L., Mitchell G.A. (2008). Lipolysis and the integrated physiology of lipid energy metabolism. Mol. Genet. Metab..

[7] Kopelman P.G. (2000). Obesity as a medical problem. Nature.

[8] Wadden T.A., Webb V.L., Moran C.H., Bailer B.A. (2012). Lifestyle Modification for Obesity. Circulation.

[9] Romijn J.A., Coyle E.F., Sidossis L.S., Gastaldelli A., Horowitz J.F., Endert E., Wolfe R.R. (1993). Regulation of endogenous fat and carbohydrate metabolism in relation to exercise intensity and duration. Am. J. Physiol. -Endocrinol. Metab..

[10] Steele J., Plotkin D., Van Every D., Rosa A., Zambrano H., Mendelovits B., Carrasquillo-Mercado M., Grgic J., Schoenfeld B.J. (2021). Slow and Steady, or Hard and Fast? A Systematic Review and Meta-Analysis of Studies Comparing Body Composition Changes between Interval Training and Moderate Intensity Continuous Training. Sports.

[11] Mercola J.M., Kirsch D.L. (1995). The Basis for Microcurrent Electrical Therapy in Conventional Medical Practice. J. Adv. Med..

[12] Iijima H., Takahashi M. (2021). Microcurrent Therapy as a Therapeutic Modality for Musculoskeletal Pain: A Systematic Review Accelerating the Translation From Clinical Trials to Patient Care. Arch. Rehabil. Res. Clin. Transl..

[13] Hamida Z.H., Comtois A.S., Portmann M., Boucher J.P., Savard R. (2011). Effect of electrical stimulation on lipolysis of human white adipocytes. Appl. Physiol. Nutr. Metab..

[14] Pano-Rodriguez A., Beltran-Garrido J.V., Hernández-González V., Reverter-Masia J. (2019). Effects of whole-body ELECTROMYOSTIMULATION on health and performance: A systematic review. BMC Complement. Altern. Med..

[15] Naclerio F., Seijo M., Karsten B., Brooker G., Carbone L., Thirkell J., Larumbe-Zabala E. (2019). Effectiveness of combining microcurrent with resistance training in trained males. Eur. J. Appl. Physiol..

[16] Noites A., Pinto J., Freitas C.P., Melo C., Albuquerque A., Teixeira M., Ribeiro F., Bastos J.M. (2015). Effects of microcurrents and physical exercise on the abdominal fat in patients with coronary artery disease. Eur. J. Integr. Med..

[17] Noites A., Nunes R., Gouveia A.I., Mota A., Melo C., Viera Á., Adubeiro N., Bastos J.M. (2015). Effects of aerobic exercise associated with abdominal microcurrent: A preliminary study. J. Altern. Complement. Med..

[18] Noites A., Moreira A., Melo C., Faria M., Vilarinho R., Freitas C., Monteiro P.R.R., Carvalho P., Adubeiro N., Amorim M. (2017). Acute effects of physical exercise with microcurrent in the adipose tissue of the abdominal region: A randomized controlled trial. Eur. J. Integr. Med..

[19] Julious S.A. (2005). Sample size of 12 per group rule of thumb for a pilot study. Pharm. Stat..

[20] Melo A.S.C., Moreira J.S., Noites A., Couto M.F., Argel Melo C. (2013). Clay body wrap with microcurrent: Effects in central adiposity. Appl. Clay Sci..

[21] Duffield R., Dawson B., Pinnington H.C., Wong P. (2004). Accuracy and reliability of a Cosmed K4b2 portable gas analysis system. J. Sci. Med. Sport.

[22] McLaughlin J.E., King G.A., Howley E.T., Bassett D.R., Ainsworth B.E. (2001). Validation of the COSMED K4 b2 Portable Metabolic System. Int. J. Sports. Med..

[23] Romijn J.A., Coyle E.F., Hibbert J., Wolfe R.R. (1992). Comparison of indirect calorimetry and a new breath 13C/12C ratio method during strenuous exercise. Am. J. Physiol. -Endocrinol. Metab..

[24] Tanaka H., Monahan K.D., Seals D.R. (2001). Age-predicted maximal heart rate revisited. J. Am. Coll. Cardiol..

[25] ACSM (2013). American College of Sports Medicine’s Guidelines for Exercise Testing and Prescription.

[26] World Health Organization (2011). Waist Circumference and Waist-Hip Ratio: Report of a WHO Expert Consultation, Geneva, 8–11 December 2008.

[27] Craig C.L., Marshall A.L., Sjöström M., Bauman A.E., Booth M.L., Ainsworth B.E., Pratt M., Ekelund U., Yngve A., Sallis J.F. (2003). International physical activity questionnaire: 12-country reliability and validity. Med. Sci. Sports Exerc..

[28] Lopes C., Aro A., Azevedo A., Ramos E., Barros H. (2007). Intake and Adipose Tissue Composition of Fatty Acids and Risk of Myocardial Infarction in a Male Portuguese Community Sample. J. Am. Diet. Assoc..

[29] Péronnet F., Massicotte D. (1991). Table of nonprotein respiratory quotient: An update. Can. J. Sport Sci..

[30] Brooks G.A. (1987). Amino acid and protein metabolism during exercise and recovery. Med. Sci. Sports Exerc..

[31] Gallagher D., Heymsfield S.B., Heo M., Jebb S.A., Murgatroyd P.R., Sakamoto Y. (2000). Healthy percentage body fat ranges: An approach for developing guidelines based on body mass index. Am. J. Clin. Nutr..

[32] Jeukendrup A.E. (2003). Modulation of carbohydrate and fat utilization by diet, exercise and environment. Biochem. Soc. Trans..

[33] Ramirez-Ponce M., Acosta J., Bellido J., Mateos J. (1998). Noradrenaline modulates the electrical activity of white adipocytes by a cAMP-dependent mechanism. J. Endocrinol..

[34] Ramírez-Ponce M.P., Mateos J.C., Bellido J.A. (2003). Human Adipose Cells Have Voltage-dependent Potassium Currents. J. Membr. Biol..

[35] Achten J., Gleeson M., Jeukendrup A.E. (2002). Determination of the exercise intensity that elicits maximal fat oxidation. Med. Sci. Sports Exerc..

[36] Blaak E.E., Saris W.H.M. (2002). Substrate oxidation, obesity and exercise training. Best Pract. Res. Clin. Endocrinol. Metab..

[37] Frayn K.N. (2010). Fat as a fuel: Emerging understanding of the adipose tissue–skeletal muscle axis. Acta Physiol..

[38] Melzer K. (2011). Carbohydrate and fat utilization during rest and physical activity. E-SPEN Eur. e-J. Clin. Nutr. Metab..

[39] Blaak E. (2001). Gender differences in fat metabolism. Curr. Opin. Clin. Nutr. Metab. Care.

[40] Bülow J., Gjeraa K., Enevoldsen L.H., Simonsen L. (2006). Lipid mobilization from human abdominal, subcutaneous adipose tissue is independent of sex during steady-state exercise. Clin. Physiol. Funct. Imaging.

[41] Carter S.L., Rennie C., Tarnopolsky M.A. (2001). Substrate utilization during endurance exercise in men and women after endurance training. Am. J. Physiol. -Endocrinol. Metab..

[42] Henderson G.C., Krauss R.M., Fattor J.A., Faghihnia N., Luke-Zeitoun M., Brooks G.A. (2010). Plasma triglyceride concentrations are rapidly reduced following individual bouts of endurance exercise in women. Eur. J. Appl. Physiol..

[43] Power M.L., Schulkin J. (2008). Sex differences in fat storage, fat metabolism, and the health risks from obesity: Possible evolutionary origins. Br. J. Nutr..

[44] Campbell S.E., Febbraio M.A. (2001). Effects of ovarian hormones on exercise metabolism. Curr. Opin. Clin. Nutr. Metab. Care.

[45] Casazza G.A., Jacobs K.A., Suh S.-H., Miller B.F., Horning M.A., Brooks G.A. (2004). Menstrual cycle phase and oral contraceptive effects on triglyceride mobilization during exercise. J. Appl. Physiol..

[46] Friedlander A.L., Casazza G.A., Horning M.A., Huie M.J., Piacentini M.F., Trimmer J.K., Brooks G.A. (1998). Training-induced alterations of carbohydrate metabolism in women: Women respond differently from men. J. Appl. Physiol..

[47] Tarnopolsky M.A. (2008). Sex Differences in Exercise Metabolism and the Role of 17-Beta Estradiol. Med. Sci. Sports Exerc..

